# Southern Surgical and Gynecological Association

**Published:** 1890-12

**Authors:** 


### SOUTHERN SURGICAL AND GYNECOLOGICAL ASSOCIATION.

THIRD ANNUAL MEETING, HELD IN ATLANTA, GEORGIA, NOVEMBER 11, 12, AND 13, 1890.

#### FIRST DAY—MORNING SESSION.

The Association convened in Concordia Hall, and was called to order by the President, Dr. George J. Engelmann, of St. Louis, Missouri, at 9:30 a. m.

Mayor Glenn delivered an address of welcome, the response to which was made by Dr. R. B. Maury, of Memphis, Tenn.

Dr. R. B. Maury, of Memphis, then contributed a paper entitled: "How Shall we Treat Our Cases of Pelvic Inflammation?"

The paper gave a comprehensive resume of the pathology of chronic pelvic inflammation as it has been clearly demonstrated by Bernutz, by Polk, Coe and others, and by the results of abdominal section. This pathology is that of pelvic peritonitis dependent upon tubal disease—not cellulitis. The author declared the term chronic cellulitis a misnomer—a pathological condition which existed only in the imagination of the physician, a term which had been productive of pernicious results in practice and which should no longer be used in connection with non-obstetric pelvic inflammation.

When the pathology rests upon such positive and abundant evidence, the question might be asked, why re-open a discussion upon it now: Because it is evident from our society proceedings and hospital reports that great confusion exists in the medical mind to-day in regard to it. Dr. Byrnes' case discussed in the New York Obstetrical Society during the present year was taken as an illustration. In speaking of such cases the great tendency to relapses in chronic pelvic inflammation was illustrated by two cases in which pus tubes were found five and seven years after attacks of peritonitis, and when it was supposed the patients were entirely restored to health. Upon the subject of treatment the writer admitted that by nonsurgical therapeutic measures large intra-peritoneal exudations are often absorbed, and even some tubal and ovarian inflammation entirely disappear and recovery seems complete. But this is the exception and by no means the rule. For the radical cure of chronic pelvic inflammation non-surgical treatment fails in a majority of the cases. A great many women suffering to a moderate degree, continue to do so in spite of the best directed non-surgical measures, and perhaps wisely elect not to undergo operation.

As a rule, the only radical and permanent relief is afforded by removal of the diseased appendages. The treatment of pus collections of course requires abdominal section.

Dr Joseph Price, of Philadelphia, followed with a paper on The Motive and Method of Pelvic Surgery. (See page 549.)

FIRST DAY—AFTERNOON SESSION.

Dr. W. H. H. Cobb, of Goldsborough, N. C., read a paper on Supra-Pubic Cystotomy in a Case of Enlarged Prostate.

The patient, a farmer, married, aged 49 years, rheumatic diathesis, dated his troubles back to 1881. While attending to the duties of Register of Deeds, he carelessly allowed overdistension of the bladder he carelessly allowed overdistension of his bladder, and had suffered more or less since that time. In 1882 he had an attack of nephritic colic and passed a small calculus, similar in size and shape to a grain of wheat. On three different occasions he passed dark, gritty deposits. In 1883 he suffered much inconvenience and some pains in urinating. In 1887 he passed a dark, gristly, bloody substance about the size of a corn-pea, accompanied by much pain and bloody urine. For the past three years he has suffered much with cystitis in a very aggrevated form, with great pain and difficulty in defecation, urine containing much blood, pus and mucus. The patient's efforts to relieve his bladder and bowels were tormenting, and night after night was spent in walking over his premises with groanings so severe as to disturb his neighbors. The patient consulted Dr. Cobb, June 15th last, and from the history of the case he suspected vesical calculus, but failed upon examination with sound to detect any stone. A digital examination, however, per rectum, disclosed the right lobe of prostate greatly enlarged, rough, indurated, exceedingly tender and sensative. Aftei consultation by letter with Hunter McGuire, he decided upon supra-pubic cystotomy as the only hope of permanent relief, which was done after the method of Dr. McGuire on June 23rd. At the expiration of two months (August 23rd,) he found the prostate perfectly normal with no symptoms of cystitis and withdrew the plug, allowing the fistula to unite, which it did in about ten days. His patient performs the act of urination and defecation without the slightest trouble, and expresses himself as entirely relieved, and is at present following his usual vocation.

Inflammation in and About the Head of the Colon.—Dr. L. S. McMurty, of Louisville, read a paper on this subject. He said the teachings to be found in systematic treatises on surgery and practical medicine upon inflammation and its results in and about the caput coli are not only worthless, but positively misleading. This is true not only as to pathology and treatment, but even as to the anatomy and relations of the caecum and its appendix.

It is a well known fact that inflammatory changes in the vermiform appendix are in almost every case the origin and seat of the inflammation diseases about the caput coli. Inflammation of the caecum is very rare, yet the testimony of surgeons and pathologists is abundant that in a certain proportion of cases caecitis, with perforation, occurs without involvment of the appendix. Regnier in 1886 operated in a case presenting symptoms of intestinal obstruction with peritonitis, doing an abdominal section. At the autopsy caecitis with perforation was discovered. In 1888, the speaker operated in case of perforative caecitis and sutured two perforations in the caecum. His patient recovered and was present in the surgical sections of the American Medical Association in May of that year.

Fecal impaction has been mentioned by surgical writers as a cause of inflammation about the head of the colon. Pain over the caecum, with a fecal mass perceptible on pressure often occurs, but rarely, if ever, associated with peritonitis. A few weeks since Dr Mc Murty saw a case in conjunction with Dr. H. H. Grant, of Louisville, in which a localized peritonitis existed, in the right iliac fossa, with a well defined firm tumor.

Abdominal section was done, and instead of appendicitis, they found the disease to be cancer of the caput coli. Irrigation and drainage rescued the patient from the immediate danger begotten by active peritonitis. The patient was a woman of middle age, and the engrafted peritonitis presented the symptoms of an acute condition. Malignant disease of the caecum has not, so far as the writer is aware, been mentioned by writers upon this subject as a probable condition in the diagnosis of deep-seated inflammation of the right iliac fossa.

The decision to operate should be determined more by the grade of the inflammation than by the time it has existed. When a diagnosis has been made. and three days have elapsed without subsidence of pulse and temperature, operation should be done.

Dr. McMurtry submitted the following conclusions:

1. Inflammation about the caput coli is, as a rule, inflammation of the appendix.
2. A certain proportion of cases will recover spontaneously by resolution. With these recurrence of the disease is common.
3. In the larger proportion the disease will endanger life, and may at any moment assume a condition practically hopeless.
4. Early operative interference involves less danger than delay, and should be resorted to in all cases in which a high grade of inflammation is persistent.
5. The essentials of the operative technique are brief anesthesia, quick and thorough work, removal of the appendix, irrigation, and drainage. The lateral incision is preferable to the median.

---

### FIRST DAY.—Evening Session.

President Engelmann delivered an address entitled: "The Causes of Ill Health in American Girls, and the Importance of Female Hygiene."

He showed that the health of the American girl is threatened and impaired by causes more or less avoidable, as they are due to our methods of life, our methods of training and education; that the physique of this girl, most favorably situated amid auspicious possibilities, is imperfect; her brain overworked, her nerve power exhausted, her function impaired, and reproduction endangered, all by reason of the susceptibility of her peculiar organization, and the increased impressionability of the sensitive system during the years of development, in which it is subjected to the most severe strain.

The remedy is attention to woman's peculiar organization and the cyclical waves of her dominant function; or, in other words, harmonious development and occupation of nerve and muscle; diminished brain-work and nerve stimulation with increased and co-ordinate physical exercise; increased protection and diminished compression of dress; self-knowledge and individual care during periods of heightened susceptibility. Changes are necessary in custom and fashion, in methods of labor and education. A harmonious co-education of mind and body should be approximated, with coincident maintenance of proper hygienic conditions.

Dr. Engelmann closed with a plea for the self-care of the American girl and her proper physiological instruction by the mother, which alone will mitigate or remove the initial cause of many of her ailments. Upon the mother he would impress that the perfect development of the female function, and the maintenace of this function, once developed, in a healthy condition, is essential to the perfect development of the girl and the perfect health of the woman; that self-care, a well regulated female hygiene, is the foundation of her well-being.

---

### SECOND DAY—MORNING SESSION.

Dr. C. A. L. Reed, of Cincinnati, Ohio, read a paper entitled, "Indications for Operation in Ectopic Gestation. (See synopsis of paper on page 560.)

Dr. Bedford Brown, of Alexandria, Virginia, followed with a paper entitled: "The Local and General Treatment of Gangrenous Wounds and Diseases."

Many years ago previous to the late war, Dr. Brown determined to institute a series of experiments to ascertain the capability of local and general treatment of all gangrenous wounds and diseases that came under his care either for their prevention or arrest. The object was to find local agents possessing active properties as stimulants of vital action in the affected parts, also as a means of disinfecting and deodorizing gangrenous sloughs, hastening their final separation and for the establishment of a healthy basis for granulation. In cases coming under his care he found that the old deodorizer failed to accomplish these objects. He then employed a solution (almost saturated) of sulphate of zinc and dilute sulphuric acid, as a local application, which seemed to meet all the requirements. The first case in which it was applied was according to the following formula:

℞. Zinci sulphatis, ℥j.
Aqua - . Oj.
Acidi sulph. dil. ℥ss.

M.

After the free application of hot water at 110 degrees the solution was applied every three hours on bats of raw cotton. In the course of two days the sloughs separated rapidly, leaving a perfectly clean, healthy basis for granulation. This solution evidently possesses active antiseptic properties. It is an admirable deodorizer; it is clean and cleanses the parts effectually. In cases of great loss of sensation in the parts, weak circulation, reduction of vital action, and depressed vitality, he knows of no agents better calculated to arouse nervous action and stagnant circulation, for as soon as the living basement is exposed it gives rise to intolerable pain. He has used this solution in all forms of gangrenous wounds and diseases, some limited, others extensive and associated with septicaemia with benefit.

Dr. Brown cited the history of several cases of different varieties of gangrenous wounds and diseases treated by various methods.

Dr. Henry F. Campbell, of Augusta, Georgia, read a paper on Vesico-Vaginal Fistulae.

Dr. W. L. Robinson, of Danville, Va., read a paper on "The Treatment of General Septic Peritonitis," in which he called attention to those cases which tended by absence of pain and a seemingly improved condition after chill and fever, to mislead as to the necessity of operating, and instanced two cases of recent date seen in consultation in which septic peritonitis and secondary abscess existed in spite of the seemingly favorable condition of the patient. He says that often there is an utter disproportion between the pathological condition and the amount of pain and tenderness, a condition so often seen in puerperal peritonitis.

He states that traumatic abdominal injuries, appendicitis and pelvic inflammations are the chief causes of septic peritonitis, while of course any internal or external influence which produces suppuration may be the indirect cause.

He agrees with Dr. G. Frank Lydston, of Chicago, that in children, falls, blows, etc.. are causes generally of peritonitis, and that because of the age of children in directing attention to the seat of injury we often diagnose the disease too late.

Dr. Robinson takes the stand that gonorrhoea is a frequent cause of septic peritonitis and the reason why it did not always produce it was, that it did not invariably invade the uterus, and even when it entered the tubes, the adhesions to the ovary rendered it self-limiting.

He holds that section, irrigation and drainage is the treatment, and that where adhesions are extensive that salines should follow the operation in order that the peristaltic action of the bowel would prevent re-formation. Cases occur which, when seen by the surgeon, are too prostrated to undergo a complete operation and the proper plan is to rapidly do what one can by section, irrigation and drainage. Dr. Robinson instanced a case of recent date in which the patient was saved when seen only *in extremis.* He urges the surgeon to go prepared to resect, anastomose, etc., as complications may indicate.

Where conditions are diagnosed which will most likely terminate in septic peritonitis, such as recurring appendicitis, that preventive measures should be undertaken: and where great tympanites exists, he would adopt Dr. Davis' mode of opening the bowel and flushing it out with hot water.

Dr. John D. S. Davis, of Birmingham, Alabama, contributed a paper entitled: "The Clinical History of the Episcystic Surgical Fistula, With Cases."

### SECOND DAY—AFTERNOON SESSION.

Dr. W. O. Roberts, of Louisville, read a paper on, "Removal of Stone from Female Bladder through the Urethra, with Cases."

This paper was devoted simply to his individual experience in the extraction through the urethra of stone from the bladder of the female. The cases thus treated were six in number; the ages of the patients ranged from 15 to 56 years. Four were married, but two only had borne children. The stones were phosphatic in four cases, uric acid in one, and an incrusted fringed body in another. In one, a very hysterical patient, the stone had for its nucleus a piece of soft wood. In one the patient had a vesico-vaginal fistula which had been closed by an operation some months prior to the occurrence of the symptoms of stone. In another the bladder had been opened by a surgecn in doing an ovariotomy upon the patient a year before the stone was discovered.

In four of the cases the stones were single, in one there were two, and in one nine. In this case the patient had passed at various times a number of small stones, from two to seven at a given micturition. These stones varied in size from that of a grain of wheat to a grain of coffee. In two years she had collected 184 stones, a number not representing all she had passed.

The extraction was done in every case, under chloroform, the patient being profoundly anaesthetized. The urethral dilatation was begun with forceps, and completed by means of the fingers. The little finger being first introduced, the ring finger next, and finally the index finger. The fingers were well oiled. In Case 1, the stone was found to be almost an inch and a half in diameter. In Case 11 the stone was found in the urethra, and proved to be a piece of soft wood heavily incrusted with urinary salts. In Case III the stone was spherical, and had a diameter of about one-half inch. In Case IV the stone was avoid, its long diameter being an inch, the shorter three-fourths of an inch. In case V there were nine stones, the smallest measuring circumambientially two and one-fourth inches; weight eighty-four grains.

Dr. William Perrin Nicholson, of Atlanta, Georgia, presented a paper entitled: "Wet Antiseptic Dressings in Injuries of the Hand."

After dwelling upon the importance of the subject, both from the standpoint of the future earning capacity of the patient, and the large amount of financial compensation demanded from corporations, he stated that for seven or eight years past he had looked after the surgery of several railroads and manufacturing establishments, and in that time had been called upon to treat more than three hundred hand injuries, representing all grades of injury from slight contusion, to complete destruction of the larger part of the hand. The especial point that was urged in the paper was the doctrine formulated by Verneuill—*never to use a scalpel in a hand injury.* The old teaching, that when a finger was crushed, you should go far enough behind the injury to secure a sound flap and amputate, was pernicious in the extreme, and had cost thousands of fingers that would have been restored to usefulness. Only such parts as were actually destroyed and pulpified should be removed, and all the tissues to come away could be amputated with the scissors. Projecting pieces of bone could be removed with plyers until reduced to the level of the fleshy parts. In compound fractures the parts should be coaptated as well as possible, and the line of separation be determined by nature, and under strict antiseptic dressings. Such a slaugh was harmless. Another point to which attention was forcibly called was the utilization of blood-clot in filling up ragged injuries, and by its substitution the restoration of lost parts. When a finger was crushed off, the end should be trimmed with scissors, and the clot utilized in building up a tissue over the bone. In reference to dressings, Dr. Nicolson said that he had tried almost all varieties, and had finally obtained the most satisfactory results from keeping the parts constantly bathed in a non-poisonous antiseptic solution.

In dealing with these wounds they were first cleansed as well as possible, and then bathed in a sublimate solution. Over all wounds a piece of aseptic rubber tissue or oiled silk was placed, then iodoform and sublimate gauze, and finally over all a covering of rubber tissue, into which, at some convenient point, a small opening was made. The patient was then given a bottle of antiseptic solution, to be carried in his pocket, if moving about, and instructed to pour, at frequent intervals, enough into this opening to saturate the dressings. He uses almost exclusively listerine, combined with a small amount of carbolic acid, in the proportion of half an ounce of the former and half a drachm of the latter, in a six ounce mixture. If there was much pain, a small amount of aqueous extract of opium was added. These dressings were not disturbed until the third day, when they were removed under strict antisepsis, to preserve the integrity of the blood-clot. Wet dressings were substituted at the end of about a week by the ordinary antiseptic dressings, kept moist by external covering of rubber tissue. Should sloughing occur, it is kept wet for a longer time with the antiseptic. Under this treatment pain was reduced to the minimum. Suppuration never occurred, and the separation of sloughs was facilitated by the warm moisture.

Dr. J. T. Wilson, of Sherman, Texas, read a paper on, "Uterine Moles and Their Treatment."

In the few cases that had come under his observation, they had been more troublesome and elicited more anxiety than most writers indicate they should, and the hemorrhages in some of the cases were alarming; then too there were some points noticed in his cases which he had failed to find described in text-books.

All authorities seem agreed upon the etiological and pathological view generally taken of it, that it is a blighted or altered conception; the ovum having perished, its coverings or the placenta, if formed when this change takes place, become attached to and continue to receive nourishment through the uterine walls and remains or becomes an organized product until it is thrown off; and this condition is attributed by some to the vitality retained in the villi of the chorion.

He had never met with a case that was lying loose in the uterus, but all were more or less adherent to its walls and most of them to the posterior wall. They had to be taken away piece-meal and the surface well curetted, washed out and carbolic acid or Churchill's iodine applied to the surface.

They all require after-treatment, because all except one case of hydatiform mole had endometritis and endocervicitis, two had severe cervical lacerations and erosions; most of them had a greater flow than usual at the subsequent menstrual periods until the inflammatory condition was relieved; in two cases the general health, while not robust, was fairly good, the others more or less delicate, none of them in perfect health, none had any history of a cancerous cachexia, nor of syphilitic taint, one was tuberculous. His experience had taught him to believe that if these cases do not receive treatment at a proper time there are two grave dangers to be apprehended, viz.: hemorrhage, which, if not an immediate cause of death, is capable of leading indirectly to that end, and septic poisoning.

In the treatment, if the cervix is sufficiently dilated and hemorrhage troublesome the mass should be promptly removed. If this cannot be done, a hot, antiseptic vaginal douche should be given, followed by a careful and efficient tampon, with the internal administration of ergot and anodynes if required, directing quiet, rest and a simple diet. In from twelve to sixteen hours the tampon should be removed and the foreign body extracted as completely as practicable; this will require a good stout pair of forceps. He had used the ordinary dressing forceps, and placental forceps, for the purpose. An excellent instrument in some cases is Emett's curette forceps. The surface should be well curetted with a wire curette, the uterus thoroughly washed out with hot solution of bichloride of mercury and Squibb's crude carbolic acid or Churchill's tincture of iodine, well applied to the surface. If much bleeding ensues—and this is not usual, the application of persulphate or perchloride of iron gives good results. The patient is put to bed and kept there as long as the indication in each special case may require; she is put upon a tonic treatment and hot vaginal antiseptic washes. In from three to five days the uterus may need curetting again and another intra-uterine douche; then the application of iodine about twice a week, alternated occasionally perhaps with carbolic acid as long as may seem necessary and the cure, if possible, completed of any uterine disease that may exist. The patient's general health is carefully looked after and her mind tranquilized.

### THIRD DAY—MORNING SESSION.

Dr. G. Frank Lydston, of Chicago, read a very elaborate and lengthy paper, entitled

### A REVIEW OF THE TREATMENT OF VARICOCELE, WITH CASES.

He said in discussing the various merits of operative procedures, it was unnecessary to take them up in detail. The *raison d'etre* of many specially devised and named operations is apparent only to the operator. For practical purposes the various methods may be divided (1) acupressure; (2) subcutaneous deligation; (3) open deligation; (4) deligation with resection of veins; (5) deligation with resection of scrotum; (6) resection of the scrotum.

The employment of acupressure, to Dr. Lydston's mind, was an evidence of a lack of faith in modern antisepsis. It reminded him of the Dutchman's method of cutting off his dog's tail, an inch at a time, so that it would not hurt him so much. Gradual obliteration of veins had all the dangers of immediate deligation in a marked degree, and had none of its advantages. The term "acupressure" covered practically all methods of gradual obliteration of the veins of which Davat's operation is an illustration. Subcutaneous deligation is not essentially dangerous in skillful hands. Simple as the operation appears, however, accidents have occurred. The operation is done in the dark, and more tissue is included in the ligature than is necessary. Strangulation of tissue is not conducive of safety. Scrotal hæmatocele, phlebitis, septic infection, thrombosis and embolism are possible. The *vas deferens* has been included in the ligature. He does not condemn the subcutaneous operation, in suitable cases and in skillful hands, but he believes there are better and safer methods on the average. There is little choice between deligation without disturbance of the veins and deligation with resection of the veins, except the remotely greater danger of sepsis in the latter. Gould's method of division by cautery he believes to be the most dangerous operation yet devised. The dangers of the open method are, in a less degree, those of subcutaneons deligation. If open ligation be determined upon, the operation should be done as high up as possible in the straight portion of the veins, and a single ligature applied to the vein. Deligation with resection of the scrotum he considers to be the ideal operation, in the majority of cases requiring surgical interference. His plan is as follows: An incision is made parallel with the spermatic cord just below the external ring. This incision should be about one inch in length. The cord is hooked out with an aneurism needle, the veins separated and tied, the ligature is cut through and the cord dropped. Sutures and antiseptic dressings complete the operation. The scrotum is now amputated by the improved Henry operation. Dr. Lydston uses decalcified bone drainage tube and juniperized silk ligatures and sutures. Resection of the scrotum he considers the simplest and safest operation for varicoceles of moderate size. In the more marked forms, the affection invariably recurs to a greater or less extent. He does not, therefore, consider the so-called Henry operation a radical cure in the true sense of the word. The author reported a number of cases operated upon by various methods, with the results, and, as far as could be learned, the subsequent history of the patient. The author had noticed hydrocele as a result of subcutaneous deligation in two cases, one operated upon by himself and the other by another surgeon. The doctor reported one very interesting case in which the scrotum was continually bathed in bloody perspiration, and in which the seminal ejaculations were heavily tinged with blood.

Dr. Willis F. Westmoreland, of Atlanta, followed wih a paper on "Morbid Reflex Neurosis."

Dr. George A. Baxter, of Chattanooga, read a paper on

SILICATE OF SODA—SOME NEW METHODS OF USE IN SURGERY, in which he said the jacket of baked silicate of soda, which he would present to the Association, possessed all the qualities to be found in the plaster, firmness and support, and weighs actually one pound and six ounces. It is neater in appearance and finish, can be perforated like leather for ventilation, which plaster cannot. It is even lighter than leather without its costly process of construction, and has the same advantage over the woven-wire jacket with the additional advantage over both these latter and all others of this class, that it can be constructed by any surgeon at any time or in any place. Dr. Baxter suspends his patient and puts roughly a plaster jacket around her, and cut this as soon as it has hardened enough to retain its shape, thereby lessening materially the time of suspension, the most trying ordeal with this or the plaster, and not without its dangers when long continued, bind the cut edges together where it has been cut down directly in front, with cords, and then place a core of paper in the center. This paper core is used for two reasons (1) to lighten the cast and take as little plaster as possible, and (2) to dry it the more readily by heating the inside. This done, the plaster is poured around the core and inside the cast, which gives him a mould of the body in extension and counter-extension, exact in every respect. Around this is made the silicate jacket after the manner of the plaster roller bandage, weaving one-half inch metal strips in the meshes of the bandage at a distance of four inches apart around the whole cast, an inside lining of a knit shirt having been first placed over the cast. The whole is then placed over a coal oil stove, and allowed to dry out, which it does in from one-half to two hours or less, especially if the cast has been previously dried. This process of heating not only dries the silicate, but bakes it as well, and renders it impervious to the action of water or the perspiration, and gives it sufficient strength to allow it being perforated for ventilation. It is now cut from the mould with a straight incision down the center, two pieces of leather, to which button hooks or eyelets have been previously attached, sewed up and down the front on each side, then the whole can be laced up solid or loosened and taken off at will. The necessity of taking off a jacket or leaving it on during the whole course of treatment will, of course, depend upon the character of the disease or injury under treatment.

Dr. Edwin Ricketts, of Cincinnati, Ohio, contributed a paper entitled: "Surgery of the Gall Bladder," in which he said to Langenbach was due the credit of totally extirpating the gall bladder, and to J. Marion Sims we owed a debt of gratitude for establishing the operation of cholecystotomy.

Dr. Ricketts reported seven cases of gall stones:

Case 1.—Mrs.——, aged 38, married, consulted him in 1880, for a tumor in her right side in the region of the gall bladder. Said she had passed by the bowel following a severe attack of hepatic colic a number of gall stones. She was emaciated and suffered from what she claimed was neuralgia of the stomach. She was slightly jaundiced and bowels constipated. Upon examination of the abdomen the tumor was well marked and nodulated above, which was the liver surface smooth. He made the diagnosis of gall stone, and urged an operation. The patient's physician, however, urged the expectant plan of treatment, which was accepted by the patient. She then went to the country, and in less than three months had an attack of hepatic colic, followed by peritonitis, dying inside of three days.

Case III.—Ellen——, colored, aged 30, consulted him for a markedly distended gall bladder, which made its appearance after a hard day's work over the wash tub. She had been sick for ten days with fever, temperature reaching 103 degrees, rapid pulse, clayish stools, with occasional attacks of hepatic colic, though not severe. He opened the gall bladder, turning out one pint of fluid which consisted of bile, mucus and pus, stitching the gall bladder up against the peritoneum. After three days catarrhal plugs were washed out of the common duct through the abdominal incision in which had been deposited a glass drainage tube. The fistulous tract is still open, discharging periodically, but with no bad results to the patient.

In Case IV, a diagnosis of cancer of the liver was made by the attending physician. The gall bladder was opened and the stone turned out, weighed 128 grains, and the common duct was filled with catarrhal deposits.

Case V.—After incising the gall bladder there escaped first about one drachm of pus, after which Dr. Ricketts turned out 23 stones. A diagnosis of cancer of the liver in this case was made by the attending physicians.

Dr. Hunter P. Cooper, of Atlanta, Ga., reported a case of "Fracture of the Femur Due to Fragilility."

Dr. Geo. H. Noble, of Atlanta, followed with an illustrative paper on "Procidentia Uteri."

---

THIRD DAY.—AFTERNOON SESSION.

Dr. W. Hampton Caldwell, of Lexington, Kentucky, read a paper on "Rectal Medication," in which he said that several years ago he was convinced of the utility and safety of rectal administration of medicine, and had ever since regarded it as a most important plan of treatment. Since we accept the theory of the local origin or manifestation of the majority of diseases, this idea of rectal administration of medicine, was more readily accepted as scientific in its applications than at any time heretofore. The rectal suppository, consisting of coco butter, incorporated with the various therapeutical agents affords the most efficient and pleasant mode of administration in our possession. Rectal suppositories satisfy all requirements as a local or constitutional remedy; they are neat, convenient, and in almost every instance preferred by the patient to the administration of the same drug by the mouth. In the administration of anodynes, it is certainly a superior method of administration to all others, as the sensitive stomach is no longer a barrier or excuse in the administration of even the most disagreeable medical agent, for we well know in many instances that this organ is either tolerant to opiates or the patient has an invincible objection to taking them, the impossibilities of the rectal administration being thrown off is one great advantage over all other methods of administration. The effects of rectal medication embrace a wide range of actions, including anodyne, antiseptic, alterant and astringent. In severe pain they certainly afford the best and safest source by which our patient's suffering can be relieved, as the action upon the rectal surface of a diffusible anodyne is quite rapid, and produces an effect about as soon as when administered by the stomach. In all inflammatory or painful affections of the abdominal or pelvic organs, this plan of administration has succeeded better than all others with the author.

Dr. Thad. A. Reamy, of Cincinnati, Ohio, reported a case in which he removed a stone weighing 365 grains, by vaginal cystotomy, from the bladder of a child six years of age, with injury of the ureter. Operations done for closing the bladder were difficult, but ultimately successful. He exhibited the stone, and made some comments on the case.

He felt after the stone was removed that it would have been better to have made supra-pubic cystotomy. Had he known the size of the stone, he would have probably done that operation. But in view of the fact that it was partly encysted, that the bladder walls were much inflamed and thickened; also the fact that in the child the parietal peritoneum dips much lower down in front of the bladder than in the adult, it became a serious question whether this course would have been better than the one pursued.

It was not clear whether the ureter was damaged in the removal of the stone, or was exposed by the sloughing which occurred much later on. He was inclined to favor the former view; and that the discharge of urine into the tissues of the bladder wall, in the line of suture, was to no small degree responsible for some of the failures in closing the bladder. However, until the last operation the most critical examination failed to discover the ureter.

Though Parvin, Campbell and others had turned an exposed ureter into the bladder, the speaker was not aware that it had heretofore been done in a subject so young. The vagina being so small, rendered the manipulation difficult in the extreme.

Dr. James A. Goggans, of Alexandria City, Alabama, read a paper on "The Surgical Treatment of Empyema."

He said during the last eighteen months he had treated six cases of empyema which developed in the wake of pneumonia, all of which had made perfect recoveries. These patients varied in age from three to thirty-five years.

Surgical treatment was the one which had been the most successfully employed. Spontaneous cures, he said, were rare—so rare that surgical interference was the rule. There were many methods of operating for the removal of pus from the pleural cavity, but they may be classified under two general headings: (1) The closed method, which consists in removing the pus by simple puncture with some kind of trocar or modern aspirator, and allowing the puncture to heal at once. (2) The open method, which consists in making an incision more or less free, with the introduction of some kind of drainage tubes to maintain the perfect evacuation of the fluid, and admit of medicated washings, and to promote free ingress and egress of air that has been passed through an antiseptic dressing. The surgical treatment, then, being an absolute necessity, we cannot over-estimate the importance of making the diagnosis certain by resorting to exploratory puncture with the hypodermic syringe. We can assure the patient and friends that no evil results can come from this procedure, and that the prognosis positively depends upon this means of settling the diagnosis.

OFFCERS FOR 1891.

President—Dr. L. S. McMurty, Louisville, Ky.
First Vice-President—Dr. J. McF. Gaston, Atlanta, Ga.
Second Vice-President—Dr. J. T. Wilson, Sherman, Texas.
Secretary—Dr. W. E. B. Davis, Birmingham, Ala.
Treasurer—Dr. Hardin P. Cochrane, Birmingham, Ala.

Place of meeting, Richmond, Virginia, second Tuesday in November, 1891.

